# Peritoneal tuberculosis and granulomatous hepatitis secondary to treatment of bladder cancer with Bacillus Calmette-Guérin

**DOI:** 10.1186/1476-0711-8-12

**Published:** 2009-04-15

**Authors:** Aliye Soylu, Ali T Ince, Hakan Polat, Nurgul Yasar, Aydin Ciltas, Selvinaz Ozkara, Ali I Tasci

**Affiliations:** 1 Department of Gastroenterology, Bakirkoy Training and Research Hospital, Istanbul, Turkey; 2 Department of Gastroenterology, Haydarpasa Numune Training and Research Hospital, Istanbul, Turkey; 3 Department of Urology, Bakirkoy Training and Research Hospital, Istanbul, Turkey; 4 Department of Internal Medicine, Bakirkoy Training and Research Hospital, Istanbul, Turkey; 5 Department of Pathology, Haydarpasa Numune Training and Research Hospital, Istanbul, Turkey

## Abstract

Intravesical administration of *Bacillus Calmette-Guérin *is used as a treatment method in superficial bladder cancer. While it is generally well tolerated, serious side effects may develop. Granulomatous hepatitis cases have been previously reported; however, only one case with tuberculous peritonitis exists in the current literature. We hereby present two cases, one of which is the second tubercular peritonitis case following *Bacillus Calmette-Guérin *treatment to be reported, and the other a case with granulomatous hepatitis. Complete cure was achieved in both cases with specific therapy. In the patient who developed peritonitis, intravesical *Bacillus Calmette-Guérin *therapy was recommenced after antituberculosis treatment, and completed without further complications.

## Introduction

*Bacillus Calmette-Guérin (BCG) *was introduced to intravesical use in high-risk superficial bladder cancer by Morales *et al. *in 1976 [1]. While the pathogenesis is unclear, it is thought to elicit a local inflammatory response [2]. Local side effects due to BCG administration are frequent (90%), and may include hematuria, and dysuria along with cystitis [3]. Various other local and systemic side effects such as acute respiratory failure and septic shock [4], hemolytic uremic syndrome, disseminated intravascular coagulation, sepsis, multi-organ failure [5,6], isolated renal tuberculosis (TB) [7], granulomatous hepatitis [8,9], peritonitis [10], pancytopenia, epididymo-orchitis [11], and diffuse granulomatous mesenteric disease [12] have also been reported. Steg *et al. *described a 3% and Lamm *et al. *around 0.7% incidence of BCG-related granulomatous hepatitis [3,13]. There exist case reports of TB peritonitis following BCG vaccination in children [14] and only one case report of peritoneal TB following the administration of BCG treatment for bladder cancer [10]. To the best of our knowledge, our patient with peritoneal TB related to intravesical BCG treatment is the second case reported so far, and for the first time initially planned BCG therapy following specific treatment of this complication was completed.

## Case report 1

A 35-years-old female patient of bladder cancer was admitted to the hospital for the second intravesical BCG administration 15 days after the first dose. She had had sudden-onset abdominal distention and pain for the last two days. The second dose of BCG to had already been postponed for a week previously due to presence of hematuria. The hematuria was considered to be secondary to the tumor since there was no biopsy or surgical intervention history in the last 30 days as well as no finding of an infection. Transurethral bladder tumor resection and biopsy were performed 1 month before. Medical history did not reveal previous TB, liver disease, or drug use. The only finding upon physical examination was extensive ascites. Laboratory tests showed aspartate aminotransferase (AST) 49 U/L (normal range (N): 5–34), alanine aminotransferase (ALT) 57 IU/L (N: 0–55), GGT: 105 (N: 9–64) IU/L, sedimentation 42 mm/h, CRP: 12 mg/dL (N: 0–5), and CA-_125_: 625 IU/ml (N: <35). Viral markers and autoantibodies were negative. Examination of the ascites fluid showed predominantly lymphocytes (85% lymphocyte, 15% PMNL) and benign mesothelial cells, with an absence of atypical cells. Glucose content was 88 mg/dl, total protein was 5.3 g/dl and albumin was 2.4 g/dl. Bacteria or acid-resistant bacilli were absent in microbiological investigation; culture and PCR assessments were negative. Adenosine deaminase (ADA) was 180 IU/L (N: 0–40). There was no pathology in the abdominal ultrasonography aside from the liver being at the upper limit of physiologically normal dimensions. Presence of BCG treatment history, >80% lymphocyte in ascites fluid, ADA> 70 IU/L and high CA-_125 _levels and exclusion of other possible conditions for differential diagnosis by further examinations led us to begin anti-TB treatment without delay due to the lack of any other explanation for the findings.

The patient was placed under treatment with three-agent therapy (isoniazid, rifampin, ethambutol) for two months and dual-agent therapy for seven months. Totally the anti-TB treatment was maintained for 9 months. AST, ALT, GGT returned to normal levels; CA-_125 _was 161 U/ml on day 15. At month three, physical examination findings and all laboratory parameters were within normal limits. Ten months after the discontinuation of therapy, BCG administration was completed to 6 doses, and there was no pathology at follow-up 2 years later.

## Case report 2

**Figure 1:**
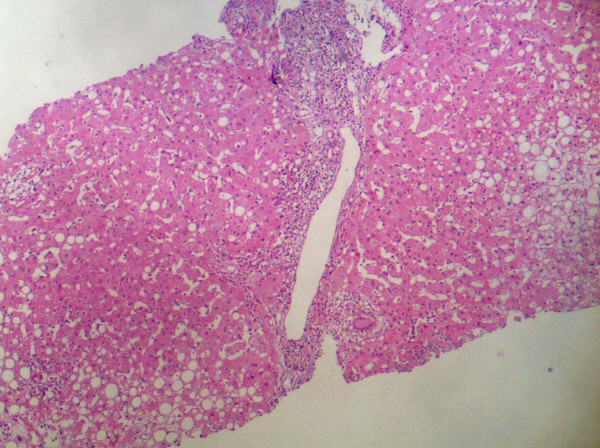
**Granuloma and lymphocytic infiltration overlapping the limiting membrane in the portal region**. Steatosis and granulomas in the liver parenchyma. (H&E×100).

**Figure 2:**
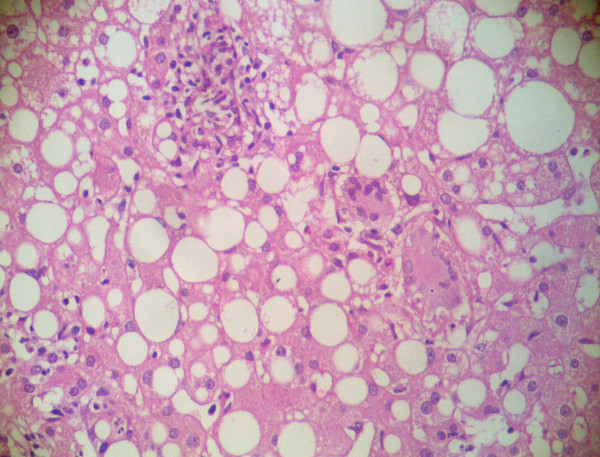
**Granulomas composed of Langhans giant cell, epithelioid histiocytosis and eosinophils in the parenchyma**. (H&E×200).

This 46-year old male patient presented with complaints of fatigue, fever and nausea following the fifth dose of intravesical BCG administration. Medical history revealed no liver disease or drug use. Pathological physical examination findings were fever (38.4°C), hypotension (85/60 mmHg), and hepatomegaly (4 cm). Laboratory tests revealed CRP: 20 mg/dL, sedimentation: 79 mm/h, AST: 137 U/L, ALT: 121 IU/L, GGT: 245 IU/L. Autoantibodies, viral markers, hemogram, and other parameters were normal. Blood culture, urine culture and PCR were negative. Liver biopsy histology showed noncaseating granulomatous hepatitis with Langhans giant cells; acid-fast bacilli stain was negative (Figure 1 and figure 2). The patient was placed under treatment with isoniazid and rifampin. On the 15th day of treatment, AST was 52 U/L, ALT: 61 IU/L, GGT: 108 IU/L. On the 20th day of therapy, ethambutol was added due to reemergence of fever and an increase in liver enzyme levels. 10 days after the initiation of three-agent therapy, 60 mg/day methylprednisolone was added due to the lack of improvement in clinical and laboratory findings. On the fourth day of corticosteroid therapy, clinical improvement and a decline in liver enzymes were observed. Steroid treatment was discontinued by gradually tapering after 1.5 months. In the third month of treatment, AST was 35 U/L, and ALT 32 IU/L. Treatment was discontinued by the sixth month of treatment. Control values one month after discontinuation were normal.

## Discussion

BCG is considered to exert an anti-tumor effect via a T-cell mediated immune response [15]. The immune system stimulation by BCG results in the detection of T-lymphocyte infiltration in the lamina propria for at least 3 months. This infiltration leads to an increase in cytotoxic T-cells and macrophages. Thus, severe local inflammation causes local ischemia and the destruction of tumor cells [16,17]. Intravesical BCG administration causes common local side effects such as hematuria, cystitis, bladder contracture, granulomatous prostatitis, and renal abscess. These are related to the inflammatory response to BCG or the contamination of the urinary system with BCG [18].

Systemic side effects are more infrequent. Factors that increase the risk of systemic side effects include bladder biopsy during BCG treatment, transurethral resection of prostate or bladder tumors, traumatic catheterization, and simultaneous cystitis [19]. Use of immunosuppressive agents, diseases such as diabetes, and genetic factors are also important risk factors [20].

The clinical symptoms of an ordinary TB peritonitis comprise 95% abdominal pain, 92% ascites and 82% abdominal distention, and culture tests are usually negative. Only three of 39 cases with TB peritonitis in the absence of BCG administration were culture-positive [21]. The culture, TB-PCR of ascites fluid and direct examination were negative in our case too. The patient was not subjected to biopsy, transurethral resection or any other invasive procedure in the last 30 days and did not have cystitis that would lead to ascites. Thus anti-TB treatment was initiated since ascites developed 15 days after the first dose of BCG and lack of any interventional procedure. Development of TB peritonitis due to BCG treatment is rarely reported [10,22]. In previously reported TB peritonitis case, ascites developed 3 months after the first dose of BCG and fever accompanied ascites development. However, in that case the patient was under peritoneal dialysis and peritoneal dialysis might well have delayed the development of ascites and the diagnosis [22]. The fluid chemistry-high ascitic ADA, ascitic fluid/serum ADA ratio and serum CA-_125 _level – was also consistent with TB peritonitis. The ascitic fluid/serum ADA ratio is higher in patients with peritoneal TB than with other causes of ascites. The optimal cut-off value for ADA was suggested to be 39 IU/L and that its sensitivity (97%) and specifity (100%) were very high in previous studies [23,24]. Serum CA-_125 _levels in patients with TB peritonitis are as high as ovarian cancers associated with peritoneal infiltration. Serum CA-_125 _levels fall between normal ranges after treatment and considered as a useful marker in the diagnosis and follow-up of patients with TB peritonitis [25,26]. The diagnosis of TB peritonitis is still difficult because of unstable presentation, low percentage of positive microscopy for acid-fast bacilli and the time delay of up to several weeks for a positive TB culture [27]. TB peritonitis may be fatal but is medically cured if diagnosed in a timely fashion. Although both non-invasive and invasive tests have additional benefits, clinical suspicion is still the first step for the diagnosis of TB peritonitis [28] and therefore empirical anti-TB treatment is indicated to patients with fluid analysis consistent with TB peritonitis if other possible causes are ruled out [29]. In the absence of a definitive treatment protocol for post-BCG TB peritonitis, we administered isoniazid, rifampin and ethambutol in the first 2 months, followed by a two-agent maintenance therapy for 7 months. Intravesical BCG treatment was reinstated nine months after treatment discontinuation, and completed to 6 doses. Certain publications do not recommend the reinstatement of BCG therapy in patients who develop systemic BCG infections [30]. In our patient, the reiteration of therapy did not cause any new pathology. Successful cure was achieved with local BCG treatment. This experience indicates that BCG treatment may be recommenced if the infection that develops after BCG administration is not considered a hypersensitivity reaction. However, continuation of intravesical BCG therapy is not recommended in cases that develop granulomatous hepatitis due to the potential of hypersensitivity reactions.

The incidence of BCG-related granulomatous hepatitis was 0.7% in a large series consisting of 2602 patients [3]. A later study by Steg *et al. *reported the incidence of serious side effects as 3%. Asymptomatic granulomas may emerge 4 to 40 months after BCG administration, while symptomatic hepatitis becomes apparent in the first few months. In the study by Steg *et al.*, four cases with BCG-related hepatitis were detected after several administrations, but one case was discovered 6 months after the completion of a two-year maintenance therapy [13]. Liver biopsy reveals granulomas in all hepatitis cases, and a direct smear for mycobacteria plays an important role. In one patient, blood culture was positive for mycobacteria after the first administration [30]. Leebeek *et al. *detected mycobacterial DNA in the liver tissue of a case with granulomatous hepatitis for the first time [31]. However, the detection of acid-fast bacilli in liver, blood, and bone marrow specimens is quite difficult. Even though acid-fast bacilli are positive in 10% of all liver tuberculosis cases, DNA hybridization studies are often negative [32]. Similar to most other cases, urine culture, blood culture, and PCR were negative in the reported cases [9,30,33].

The development of BCG-related granulomatous hepatitis is alleged to be a hypersensitivity reaction to the protein fraction of BCG [34]. Granulomatous hepatitis cases that are not responsive to anti-TB drugs and corticosteroids exist [33,35]. The treatment of these cases always involves steroids together with anti-tuberculosis therapy [34]. Hypersensitivity and infection cannot be differentiated histopathologically and clinically; anti-TB agents and steroids can be co-administered until culture tests are finalized [30,31,33,35]. No definitive treatment regimen has been established for BCG-related granulomatous hepatitis, but early treatment is recommended [3,30,32]. The recommended first-line therapy for granulomatous hepatitis is isoniazid plus rifampin. Clinical progress is monitored by the decline and normalization of liver function tests. If signs of a hypersensitivity reaction are present, steroids are added [30]. The addition of prednisolone to the treatment protocol in patients with elevated liver function tests despite a 6-month therapy with rifampin and isoniazid resulted in an improvement in laboratory and histological tests. Rapid response to therapy is a clinical sign that supports hypersensitivity [33]. The recommended therapy for severe systemic BCG infection is the administration of isoniazid, rifampin and ethambutol for six months [3,13,32]. Treatment with pyrazinamide is not recommended, as all forms of *M. bovis *are resistant [30]. While some investigators recommend prophylactic therapy with 300 mg isoniazid daily, others suggested that this might diminish the anti-tumor effect of BCG [3]. On the other hand a more recent study failed to find any evidence of either benefit or harm in giving prophylactive INH and prophylactive ofloxacin was beneficial in reducing moderate-severe side effects [36,37].

Even in the absence of traumatic administration, patients should be monitored for side effects during BCG treatment. Liver function tests must be monitored during BCG treatment. BCG-related granulomatous hepatitis should be considered in cases of abnormal liver function tests and persistent fever following BCG therapy. In cases with an appropriate clinical presentation, negative culture tests should not be a cause for treatment delay. It should be remembered that early treatment improves the chance of success.

## Abbreviations

ALT: alanine aminotransferase; AST: aspartate aminotransferase; BCG: Bacillus Calmette-Guérin; H&E: haematoxylin and eosin; TB: tuberculosis; ADA: adenosine deaminase.

## Competing interests

The authors declare that they have no competing interests.

## Authors' contributions

AS planned and coordinated the study, and prepared manuscript; ATI was involved in data entry; HP, NY and AC were involved in literature search; SÖ prepared and stained specimens, and evaluated specimens; AIT was involved in urological treatment and follow-up of the patient. All authors read and approved the final manuscript.
